# Checkpoint inhibitors create rogue regulatory T cells

**DOI:** 10.1172/JCI206592

**Published:** 2026-07-01

**Authors:** Smriti Parashar, Klaus Ley

**Affiliations:** Immunology Center of Georgia, Augusta University, Augusta, Georgia, USA.

## Abstract

Immune checkpoint inhibitor–induced inflammatory arthritis (ICI-IA) is an immune-related adverse event (irAE) following treatment with PD-1, PD-L1, or CTLA-4 inhibitors in patients with cancer. In this issue of the *JCI*, Ma and colleagues identified a subset of regulatory T cells (Tregs) that coexpress CD137 and IL-6 receptor (IL6R), termed atypical Tregs (AtpTregs), which are selectively enriched in patients with ICI-IA. Functionally, AtpTregs exhibited reduced suppressive capacity and a Th17-like proinflammatory phenotype. Notably, these cells were associated with more severe arthritis, yet improved cancer outcomes, suggesting a potential role in tumor control. The anti-IL6R therapy tocilizumab, administered as an off-label intervention for ICI-IA, reduced AtpTreg abundance and alleviated arthritis while maintaining antitumor immunity in a small cohort of patients with new-onset ICI-IA. Thus, anti-IL6R could be a targeted approach to manage ICI-IA and potentially other irAEs involving AtpTregs.

## Drivers of immune-related adverse events during cancer immunotherapy.

Immune checkpoint inhibitors (ICI) have emerged as a breakthrough in cancer therapy, activating the immune system against tumors by blocking inhibitory checkpoint cell surface receptors such as CTLA-4 and PD-1 or PD-L1 (1). However, a major side effect of ICI therapy is the development of immune-related adverse events (irAEs), which are characterized by loss of self tolerance and consequent immune-mediated damage to nontumor tissues (2). ICI-induced inflammatory arthritis (ICI-IA) is an irAE that affects 5%–7% of patients, causing considerable pain, joint impairment, and occasionally necessitating discontinuation of ICI therapy (3). Identifying the key drivers of ICI-IA is therefore critical for developing strategies that alleviate arthritis symptoms without compromising antitumor responses in these patients.

**Figure 1:**
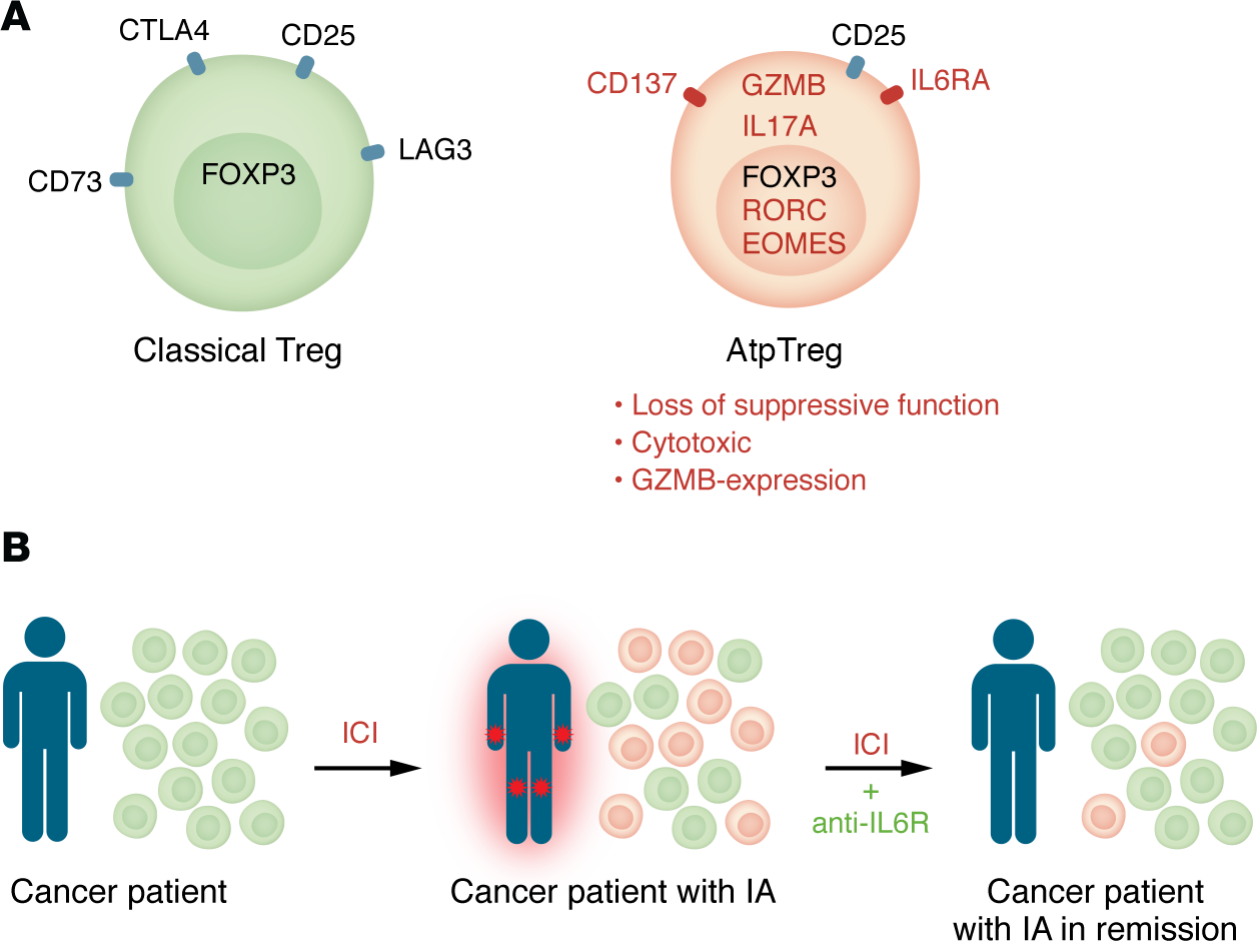
ICI-IA is associated with emergence of atypical Tregs that are sensitive to IL6R inhibitors. (**A**) Ma et al. (4) showed that a distinct subset of atypical regulatory T cells (AtpTregs, tan) is enriched in patients treated with immune checkpoint inhibitors (ICIs) who developed ICI-induced inflammatory arthritis (ICI-IA). These AtpTregs retained the expression of classic Treg (blue) markers like CD25 and FOXP3 and were identified by the surface expression of CD137 and IL6 receptor (IL6RA). AtpTregs lost suppressive function and lost Treg markers like CTLA4, CD73, and LAG3. Instead, they gained the Th17-lineage markers RORC and IL17A. AtpTregs were cytotoxic and expressed granzyme B (GZMB). (**B**) Treatment of patients with ICI-IA with the anti-IL6R mAb tocilizumab reduced the number of AtpTregs and improved arthritis outcomes.

In this issue of the JCI, Ma et al. (4) reported that ICI-IA is associated with a unique CD137^+^ IL-6 receptor α (IL6RA)^+^ subset of regulatory T cells (Tregs), which they refer to as atypical Tregs (AtpTregs). AtpTregs were significantly enriched in ICI-IA–treated patients compared with patients whose cancer was comorbid with rheumatoid arthritis (CA-RA) or ICI-treated patients who did not experience an irAE. Compared with Tregs, AtpTregs displayed reduced suppressive capacity, expressed IL-17, and exhibited an inflammatory phenotype (Figure 1A). Their frequency was positively correlated with ICA-IA but also with overall survival, suggesting that they may be involved in tumor control.

## Implicating Treg-derived subpopulations in ICI-IA pathology.

Treg infiltration in tumors is generally associated with adverse clinical outcomes (5). As Tregs predominantly recognize MHC class II-restricted self epitopes (6) and tumors can express self antigens, one function of ICIs may be to overcome the regulatory quality of Tregs. This would explain both the antitumor effects and the autoimmune side effects of ICI treatments.

To better characterize AtpTregs and assess their cross-tissue features, the authors profiled Tregs from peripheral blood, synovial fluid, and tumor samples from ICI-treated patients by single-cell RNA sequencing (scRNA-Seq) with antibody sequencing (Ab-Seq). AtpTregs of ICI-IA patients exhibited an activated effector-memory phenotype and expressed ICOS, CD40L, PD-1, and CD137. They also expressed the T-helper (Th)17 markers IL-17 and RORC and the cytotoxic marker granzyme B (GZMB). A similar but less characterized population of atypical Tregs expressing CD137 and ICOS was recently identified in the synovial fluid of patients with ICI-IA (7).

Treg-derived cells have previously been described in mouse models of cancer (8, 9). The conversion of Tregs to other phenotypes is variably described as plasticity or instability, ultimately leading to a loss of the Treg lineage-defining transcription factor FOXP3. Such cells have previously been called exTregs (10, 11). Some human exTregs are cytotoxic, as evidenced by their expression of GZMB and perforin (12, 13). In mice, follicular helper exTregs (14), Th17 exTregs (10), and Th1 exTregs (15, 16) have also been described.

It seems like the AtpTregs characterized by Ma et al. (4) are not exTregs but may represent an earlier stage of Treg instability, because they express normal levels of FOXP3 and near-normal levels of the IL-2 receptor CD25. Additionally, coexpression of RORC and EOMES suggests that these cells may represent a hybrid, plastic Treg population with features of both Th17 and cytotoxic/effector T cell lineages. A Th17 subset of CD4^+^ T cells has previously been shown to be enriched in the synovial fluid of patients with ICI-IA (17). The reduced suppressive capacity of AtpTregs suggests that functional impairment in pathogenic Treg subsets may precede, or occur independently of, loss of FOXP3 expression.

Single-cell VDJ-T cell receptor (TCR) sequencing across multiple compartments (blood, synovial fluid, and tumor) in patients with ICI-IA revealed that nearly all expanded clones were specifically enriched in AtpTregs, but not in other Treg subtypes. Moreover, these expanded clonotypes were shared across compartments, potentially suggesting inter-tissue trafficking. However, the specific antigens driving AtpTreg expansion remain unknown. Ma et al.’s longitudinal investigation of eight patients before and after ICI treatment suggests that AtpTregs do not arise immediately after ICI administration but emerge specifically at ICI-IA disease onset. This is interesting, because it implies that AtpTregs are not a direct, immediate consequence of checkpoint blockade but instead arise in response to immune dysregulation associated with ICI-IA. This highlights their possible role as both mediators of ICI-IA and as an early biomarker for disease onset.

## Tocilizumab intervention reduces AtpTregs and improves ICI-IA.

To assess the therapeutic relevance of their findings, the authors analyzed an independent cohort of 18 patients with ICI-IA treated with the anti-IL6R antibody tocilizumab. Treatment with tocilizumab led to significant improvement in arthritis, as measured by objective parameters (serum CRP levels, MRI imaging) and patient-reported quality-of-life scores. This was accompanied by a reduction in AtpTregs in both synovial fluid and peripheral blood, suggesting that AtpTregs may drive the arthritis phenotype. Transcriptional profiling of all Tregs after tocilizumab treatment revealed increased expression of suppression markers (CTLA4, IL10) and decreased expression of proinflammatory and cytotoxic mediators (IL17A, GZMB). Th17 scores consistently decreased while Treg scores increased. Concomitantly, Treg suppressive function was improved. Importantly, Kaplan-Meier analysis indicated no significant difference in overall survival in tocilizumab-treated versus untreated patients with ICI-IA, suggesting that IL6R blockade may be a practical option for patients with ICI-IA (Figure 1B).

## Outstanding questions and implications for understanding ICI-driven autoimmunity.

Several limitations exist that open doors for future studies. First, the sample sizes in Ma et al.’s patient cohorts are relatively small, which may limit the generalizability of the results. Second, the study does not define if appearance of AtpTregs is specific to PD-1 or PD-L1 blockade or can be extended to other checkpoint inhibitors. It is not known whether AtpTregs only appear in ICI-IA or are also common in other irAEs like colitis, hepatitis, or myocarditis. Third, whether AtpTregs arise from conventional Tregs, represent a distinct lineage, or are induced from other CD4^+^ T cell populations remains unclear. Fourth, the stability of AtpTregs over time and their potential progression to fully destabilized exTregs remains to be addressed. Finally, although no survival differences were observed following tocilizumab treatment in this small cohort, larger studies across diverse cancer types are needed to confirm its efficacy and safety to treat irAEs.

Beyond its immediate therapeutic implications, this work highlights that irAEs may result not only from heightened immune activation but from qualitative shifts in immune cell identity, particularly within Tregs. This emphasizes the importance of gaining a deeper mechanistic understanding of the pathways driving Treg plasticity, especially in the context of ICI therapy. Future research can determine how prevalent AtpTreg-mediated autoimmunity is among patients receiving ICIs across multiple cancer types. If confirmed, targeting these cells could be a valid strategy to improve quality of life without compromising antitumor therapy in such patients.

## Conflict of interest

The authors have declared that no conflict of interest exists.

## Funding support

This work is the result of NIH funding, in whole or in part, and is subject to the NIH Public Access Policy. Through acceptance of this federal funding, the NIH has been given a right to make the work publicly available in PubMed Central.

- Awards P01 HL136275 to Catherine C. Hedrick (project 4 PI Klaus Ley) and R35 HL145241 to KL.

## Version 1 07/01/2026

Electronic publication
